# HIOPP-6 – a pilot study on the evaluation of an electronic tool to assess and reduce the complexity of drug treatment considering patients’ views

**DOI:** 10.1186/s12875-022-01757-0

**Published:** 2022-06-28

**Authors:** Viktoria S. Wurmbach, Steffen J. Schmidt, Anette Lampert, Simone Bernard, Andreas D. Meid, Eduard Frick, Michael Metzner, Stefan Wilm, Achim Mortsiefer, Bettina Bücker, Attila Altiner, Lisa Sparenberg, Joachim Szecsenyi, Frank Peters-Klimm, Petra Kaufmann-Kolle, Petra A. Thürmann, Hanna M. Seidling, Walter E. Haefeli

**Affiliations:** 1 Department of Clinical Pharmacology and Pharmacoepidemiology, Heidelberg University Hospital, Im Neuenheimer Feld 410, 69120 Heidelberg, Germany; 2 Cooperation Unit Clinical Pharmacy, Heidelberg University Hospital, Im Neuenheimer Feld 410, 69120 Heidelberg, Germany; 3 Department of Clinical Pharmacology, School of Medicine, Faculty of Health, Witten/Herdecke University, Alfred-Herrhausen-Straße 50, 58448 Witten, Germany; 4 Institute of General Practice (Ifam), Centre for Health and Society (Chs), Medical Faculty, Heinrich Heine University Düsseldorf, Moorenstr. 5, 40225 Düsseldorf, Germany; 5 Present Address: Professorship of Primary Care, Faculty of Health, Witten/Herdecke University, Alfred-Herrhausen-Straße 50, 58448 Witten, Germany; 6 Institute of General Practice, Rostock University Medical Center, Doberaner Straße 142, 18057 Rostock, Germany; 7 Department of General Practice and Health Services Research, Heidelberg University Hospital, Im Neuenheimer Feld 130.3, 69120 Heidelberg, Germany; 8 AQUA-Institute for Applied Quality Improvement and Research in Health Care, Maschmühlenweg 8–10, 37073 Göttingen, Germany; 9 Philipp Klee-Institute for Clinical Pharmacology, HELIOS Clinic Wuppertal, Heusnerstraße 40, 42283 Wuppertal, Germany

**Keywords:** Drug treatment, Complexity factor, General practice, Patient-centered care, Shared decision making

## Abstract

**Background:**

A complex drug treatment might pose a barrier to safe and reliable drug administration for patients. Therefore, a novel tool automatically analyzes structured medication data for factors possibly contributing to complexity and subsequently personalizes the results by evaluating the relevance of each identified factor for the patient by means of key questions. Hence, tailor-made optimization measures can be proposed.

**Methods:**

In this controlled, prospective, exploratory trial the tool was evaluated with nine general practitioners (GP) in three study groups: In the two intervention groups the tool was applied in a version with (G_I_with_) and a version without (G_I_without_) integrated key questions for the personalization of the analysis, while the control group (G_C_) did not use any tools (routine care). Four to eight weeks after application of the tool, the benefits of the optimization measures to reduce or mitigate complexity of drug treatment were evaluated from the patient perspective.

**Results:**

A total of 126 patients regularly using more than five drugs could be included for analysis. GP suggested 117 optimization measures in G_I_with_, 83 in G_I_without_, and 2 in G_C_. Patients in G_I_with_ were more likely to rate an optimization measure as helpful than patients in G_I_without_ (IRR: 3.5; 95% CI: 1.2—10.3). Thereby, the number of optimization measures recommended by the GP had no significant influence (*P* = 0.167).

**Conclusions:**

The study suggests that an automated analysis considering patient perspectives results in more helpful optimization measures than an automated analysis alone – a result which should be further assessed in confirmatory studies.

**Trial registration:**

The trial was registered retrospectively at the German Clinical Trials register under DRKS-ID DRKS00025257 (17/05/2021).

**Supplementary Information:**

The online version contains supplementary material available at 10.1186/s12875-022-01757-0.

## Introduction

A complex drug treatment results from various factors of a drug treatment or the medication process, that potentially make the administration of a drug treatment difficult for patients. Many of these so-called complexity factors are associated with non-adherence or administration errors [1], suggesting that they are also linked to treatment success. However, a distinct definition of a complex drug treatment is still missing so far [1, 2]. Different approaches have been described to assess and reduce complexity of drug treatment. For example, the established Medication Regimen Complexity Index (MRCI), a score to assess the complexity of a medication regimen [3], has been automated to identify patients with a complex medication regimen. This algorithm was combined with the proposal of standardized optimization measures to nurses [4, 5]. Another approach was to develop guiding questions to help health professionals reduce the complexity of drug treatment [6].

All currently available approaches address health professionals, but do not consider individual patient perspective. However, it is well known that patient centeredness is essential to effectively tailor health care to patient needs, thereby fostering the acceptance and sustainability of potential changes in drug treatment [7] while promoting patient adherence and satisfaction at the same time [8–10].

Therefore, an electronic tool was developed that is applied by health professionals but attempts to comprehensively consider the patient perspective [11]. It automatically screens structured medication data for 38 known factors contributing to complexity (automated assessment). Subsequently, the tool personalizes the results by evaluating the actual relevance of the factors identified for the patient by proposing key questions for the health professional using the tool. These key questions address the complexity factors identified in the automated assessment in order to evaluate whether the patient indeed experiences difficulties in this respect and therefore needs help. Based on the respective patient’s response, specific optimization measures are proposed that have the potential to eliminate the complexity factor (e.g. by exchanging the drug or changing the dosage scheme) or to mitigate its impact (e.g. by offering teaching material to the patient). Furthermore, the tool displays eight additional questions on 14 possible problems with drug handling and administration (e.g. potential problems with swallowing) the health professional should ask every patient.

The objective of this pilot study was to evaluate the benefit of the developed tool in primary care. It was hypothesized that optimization measures recommended to the patient by the health professional are more often considered helpful by the patient when based on an automated and personalized complexity analysis compared to an exclusively automated complexity analysis.

## Methods

### Study design

**Fig. 1:**
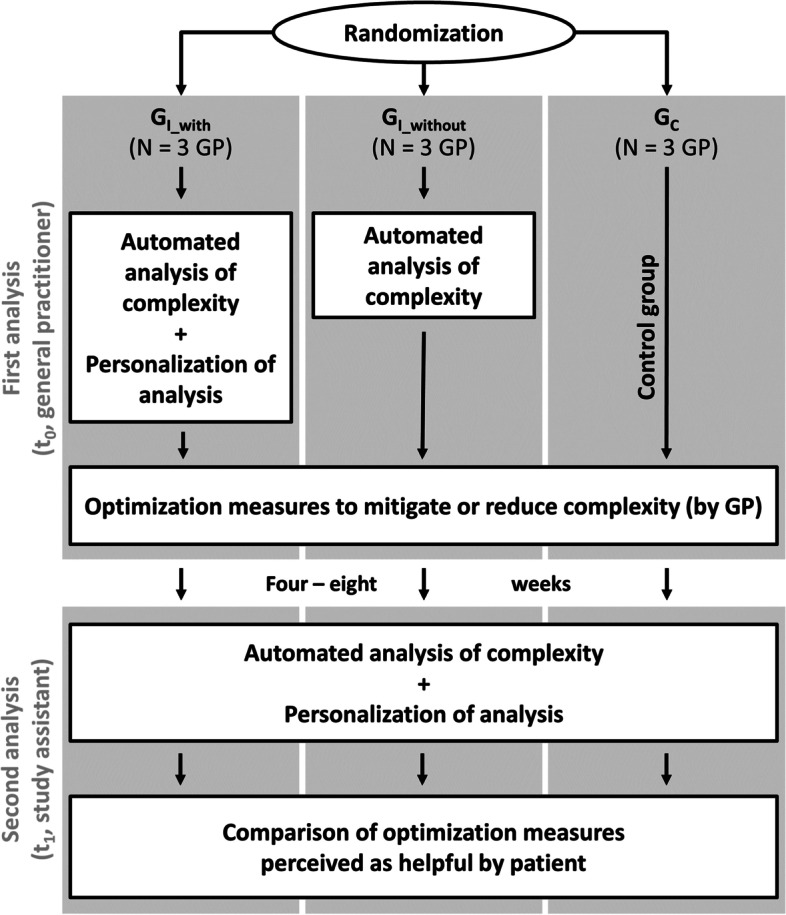
Study design

The tool was exploratively evaluated in a controlled, prospective, pilot study with general practitioners (GP) allocated to three study groups. Group 1 (G_I_with_) used the full version of the tool [11], while group 2 (G_I_without_) used a version of the tool with limited functionality that did not propose key questions and, therefore, lacked personalization of the automated results. Group 3 (G_C_) was a control group representing routine care where physicians did neither receive a training in complexity reduction nor use any version of the tool. In each group, complexity was assessed at two points in time (Fig. 1): the first analysis (t_0_) was performed by the GP either as part of a regular patient consultation, or at a specifically arranged appointment (depending on the preference of GP and patients), the second analysis was performed by a study assistant in person or by telephone and was planned four weeks after t_0_. The study was conducted from May to October 2018 after receiving unrestricted ethical clearance (i.e. from the Medical Faculty of Heidelberg (S-672/2017), the University of Witten/Herdecke (57/2018), and the respective responsible authorities of the practice locations). The study was carried out in context of the Declaration of Helsinki and the data protection laws of the German state and the respective federal states as well as the European General Data Protection Regulation (GDPR), which came into force during the course of the study. The trial was registered retrospectively at the German Clinical Trials register under DRKS-ID DRKS00025257 (17/05/2021).

### GP recruitment and randomization

For inclusion GP had to meet the following criteria: routine use of the nationally standardized medication schedule (“Bundeseinheitlicher Medikationsplan”, BMP) and access to the internet. Nine GP were recruited in three different German federal states (Baden-Wuerttemberg: *N* = 4, North Rhine-Westphalia: *N* = 3, Mecklenburg-Western Pomerania: *N* = 2). Among the GP of each federal state one was randomly assigned to one of three groups by the study team using the algorithm at www.randomization.com (first generator). This way each study group was supposed to contain one GP of each federal state, but one GP withdrew study participation and was replaced (eventually two GP of G_I___without_ were located in the same federal state). Before inclusion of patients, all GP were trained in the study protocol and the GP allocated to G_I_with_ and G_I_without_ received a face-to-face training in the use of the respective tool version.

### Patient recruitment and inclusion

Patients were recruited non-consecutively in each general practice and each practice should include approximately 20 patients. Given the explorative nature of the study, sample size per practice was determined based on previous experiences of the study team concerning the feasibility of patient recruitment. For inclusion, patients had to be at least 18 years old and to give written informed consent. Furthermore, patients had to use more than five drugs by themselves and on a long-term basis. Polypharmacy is a complexity factor that was assumed to be identified easily by the GP and, thus, every patient was affected by at least one complexity factor in the medication regimen. Patients were excluded if poor language skills or cognitive or physical impairment prevented them from participation or if they withdrew their consent. Patients of all groups were asked to fill in a paper-based questionnaire with their sociodemographic data before the analysis. Thereby, additional patient characteristics assumed to potentially affect patient’s perception of the study were collected (e.g. a prior medical knowledge or difficulties in medication administration experienced in the past).

### First analysis (t_0_)

In G_I_with_ and G_I_without_, GP analyzed the current drug treatment with the respective tool version and consequently took measures to mitigate or reduce the complexity. The selection of the optimization measures, the communication of the measures (including verbal information and potentially handout of the provided patient leaflets) and eventually also the implementation was left to the responsibility and discretion of the GP. Thereby, GP could refer to the optimization measures suggested by the electronic tool or also implement their own measures. The drug regimen, all complexity factors identified, the time required for the analysis, and, if applicable, answers to key questions as well as the optimization measures chosen were documented in the tool by the GP (respective buttons were integrated in the tool). Additionally, GP could also document whether they carried out an optimization measure that was not suggested by the tool. Subsequently, GP in G_I_with_ and G_I_without_ were asked by the tool, whether they believed that the analysis had simplified drug treatment for the respective patient or not. The GP of G_C_ did not receive any support for analyzing and reducing complexity but were aware of the study´s focus and, thus, could have concentrated on the complexity of drug treatment as part of the routine patient consultation. The BMP of patients of G_C_ was analyzed retrospectively with the electronic tool by the study team.

### Second analysis (t_1_)

The current drug treatment was reanalyzed by a study assistant (pharmacist). For this purpose, the study assistant obtained copies of the patients’ BMPs from the GP practices, which, if necessary, had been updated by the GP after the first analysis. The study assistant analyzed the BMPs of all patients with the full version of the electronic tool (automated and personalized complexity analysis) in all study groups. Furthermore, the patients in G_I_with_ and G_I_without_ were asked to rate if the optimization measures recommended by the GP at t_0_ have had an influence on the handling of their drug treatment (four-point Likert item: improved, little improved, no effect, worsened). Thereby, the wording should indicate on whether the measures were indeed implemented by the patient. Conversely, patients of G_C_ were asked whether the GP made any recommendations, gave any explanations or adapted the medication schedule in order to address the complexity of their drug treatment. If changes in the BMP were noticed at t_1_ that were not documented in the tool, the patients were asked whether or not they were made by the GP based on the initial complexity analysis (G_I_with_ and G_I_without_) respectively the study visit (G_C_).

### Outcome

Primary outcome was the number of optimization measures per patient reported as helpful by patients. Counting absolute numbers was considered more suitable than for instance a percentage, because the use of percentages would have overestimated the ratings of patients with only small numbers of optimization measures proposed. Optimization measures were considered not helpful if patients indicated at t_1_ that it did not improve (no effect) or even worsened the handling of their drug treatment. Furthermore, optimization measures that patients could not remember at t_1_ were considered not helpful. The number of complexity factors and medications and the type and number of recommended and helpful measures were analyzed in all three groups. The time needed for the analysis with the electronic tool was evaluated in groups G_I_with_ and G_I_without_.

### Statistical analysis

All results as well as the socio-demographic data were reported descriptively (mean and standard deviation, or median and minimum/maximum). The primary outcome was compared between G_I_with_ and G_I_without_ using Poisson regression; incidence rate ratios (IRR) were derived as exponentiated parameter estimates from the regression model. All other outcomes and socio-demographic data were analyzed using nonparametric tests (i.e. χ^2^-test, Kruskal–Wallis test, Mann–Whitney *U* test). P-values less than 0.05 were considered as formally significant, yet all p-values should be regarded as exploratory. All analyses were carried out using Microsoft Excel 2016, IBM SPSS Statistics 25 and the R software environment in version 4.0.2 (R Foundation for Statistical Computing, Vienna, Austria).

## Results

### Patient characteristics

**Fig. 2:**
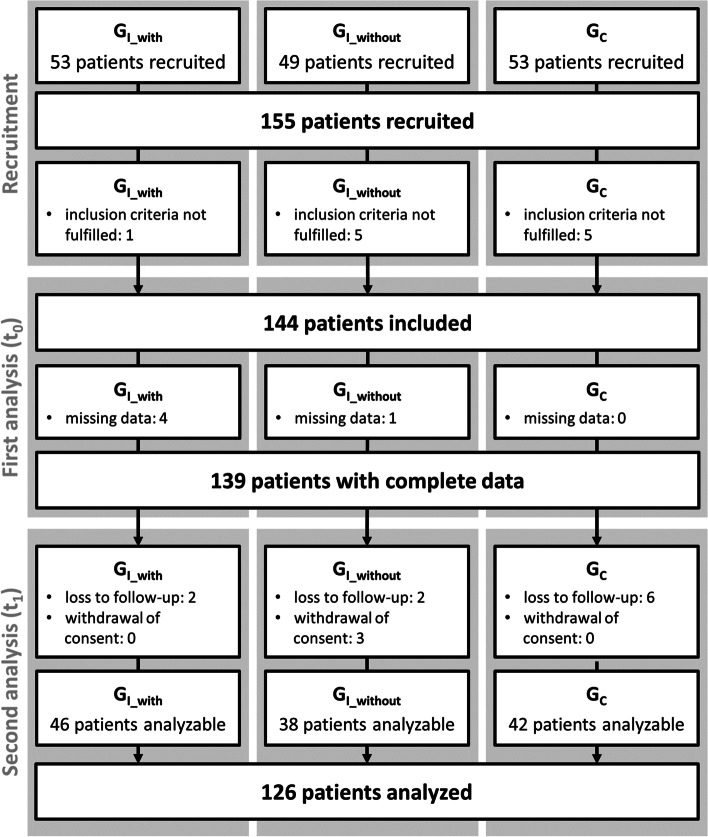
Recruitment and inclusion of patients

A total of 155 patients were recruited, 144 patients met the inclusion criteria, for 139 patients complete data at t_0_ were available, and for 126 patients (G_I_with_: 46; G_I_without_: 38; G_C_: 42) complete data on both visits were collected, allowing these patients to be included for statistical analysis (Fig. 2). The groups were comparable in socio-demographic details (Table 1), with the exception of the mean duration of GP-patient-relationship.

**Table 1:** Socio-demographic data (collected via paper-based questionnaire)

	**G**_**I_with**_ (*N* = 46)	**G**_**I_without**_ (*N* = 38)	**G**_**c**_ (*N* = 42)
Age, mean ± SD [years]	71.6 ± 11.1 (m.a.: 1)	73.9 ± 9.4 (m.a.: 2)	70.3 ± 11.6
Number of women (%)	21 (45.7)	17 (44.7) (m.a.: 1)	21 (50.0)
Number of medications, mean ± SD	9.9 ± 2.6	8.9 ± 2.1	9.1 ± 2.6
Number of patients having the following education (%)			
no graduation	2 (4.3)	1 (2.6)	3 (7.1)
lower secondary	29 (63.0)	27 (71.1)	18 (42.9)
secondary	5 (10.9)	4 (10.5)	10 (23.8)
high school	8 (17.4)	4 (10.5)	8 (19.0)
other	1 (2.2)	1 (2.6)	3 (7.1)
missing answer	1 (2.2)	1 (2.6)	0 (0)
Number of patients having medical knowledge because of their profession (%)	4 (8.7)	3 (7.9) (m.a.: 2)	0 (0) (m.a.: 5)
Median duration of GP-patient-relationship [years] (range)	9* (0.5 – 45.0) (m.a.: 3)	16* (1 – 40.0) (m.a.: 3)	5* (0.2 – 35.0) (m.a.: 2)
Number of patients who experienced difficulties with medication administration in the past (%)	5 (10.9)	5 (13.2) (m.a.: 1)	7 (16.7)

^*^Statistically significant difference (*P* = 0.001; Kruskal–Wallis test); G_I_with_: automated and personalized analysis; G_I_without_: exclusively automated analysis; G_C:_ routine care; m.a.: missing answer

The actual interval between the first and the second analysis was 7.1 ± 2.1 weeks, 6.4 ± 1.8 weeks, and 6.1 ± 1.2 weeks in G_I_with_, G_I_without_, and G_C_, respectively, with no significant differences between the groups (*P* = 0.107; Kruskal–Wallis test). In G_C_ the date of the written informed consent was used for this calculation, assuming that written informed consent was obtained at the date of t_0_.

### Number of complexity factors and number of medications

There was no significant decrease in the number of complexity factors detected automatically in the BMPs from t_0_ to t_1_ in any group. Furthermore, the number of complexity factors did not differ significantly between the groups at t_0_, but it did at t_1_ (Table 2). There was no significant difference in the number of medications at t_0_ and t_1_ for each group or between the groups at any time of analysis (see Additional File 1).

**Table 2:** Number of drugs and automatically detected complexity factors per patient, for all groups and both time points respectively

	G_I_with_	G_I_without_	G_C_	Between-group comparison^a^
Drugs				
Median number of drugs at t_0_ (range)	9.5 (6—15)	8.5 (6—14)	9.0 (6—16)	*P*_ANOVA_ = 0.284
Median number of drugs at t_1_ (range)	9.0 (5—18)	8.5 (6—14)	8.0 (6—16)	*P*_ANOVA_ = 0.148
Within-group comparison (t_0_ vs t_1_)^a^	*P*_ANOVA_ = 0.921	*P*_ANOVA_ = 0.939	*P*_ANOVA_ = 0.715	
Complexity factors				
Median number of factors at t_0_ (range); total number of factors	7.0 (1—20); 360	6.0 (1—19); 260	6.0 (1—22); 286	*P*_ANOVA_ = 0.134
Median number of factors at t_1_ (range); total number of factors	6.5 (0—16); 336	6.0 (1—18); 239	5.0 (1—21); 252	*P*_ANOVA_ = 0.044
Within-group comparison (t_0_ vs t_1_)^a^	*P*_ANOVA_ = 0.363	*P*_ANOVA_ = 0.347	*P*_ANOVA_ = 0.143	

^a^Poisson regression with an ANOVA-based group comparison (test statistics compared by chi-square distribution); analysis of number of drugs was controlled for number of factors and vice versa

### Time required for analysis

There was no significant difference (*P* = 0.718; Mann–Whitney *U* test) for the average time required between the groups G_I_with_ (6.4 ± 3.6 min) and G_I_without_ (6.3 ± 4.3 min).

### Type and number of optimization measures

**Fig. 3:**
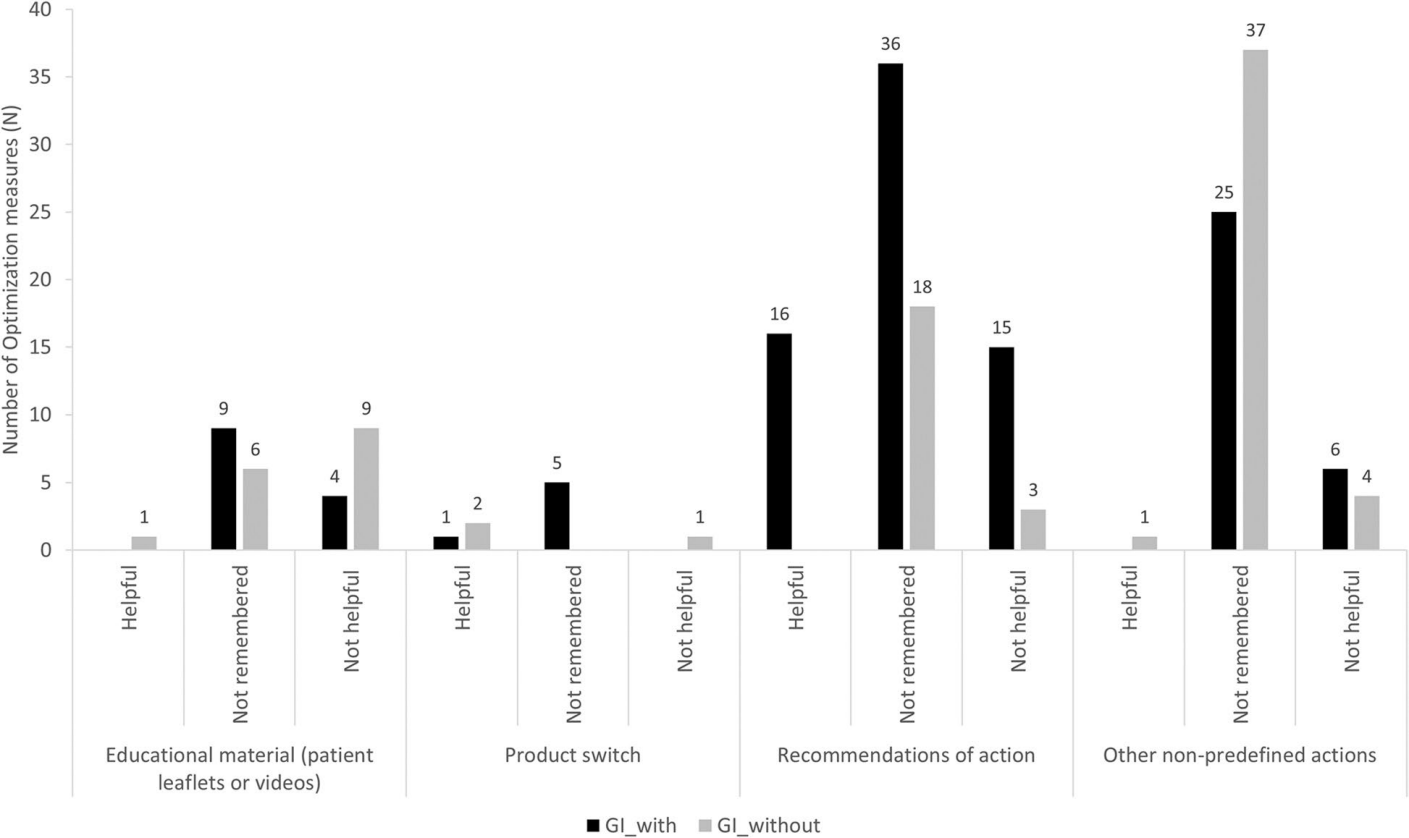
Type of optimization measures recommended by GP in G_I_with_ (*N* = 117) and G_I_without_ (*N* = 83; one optimization measure could not be analyzed) and their evaluation by patients. GI_with: automated and personalized analysis; GI_without: exclusively automated analysis; Not remembered: Patient could not remember an optimization measure or patient was sure that he or she had not received the optimization measure or patient was unsure whether he or she had received it

There was a significant yet very small difference in the counts of optimization measures that were considered helpful by patients (Fig. 3), between G_I_with_ (17 of 117 recommended measures; on average 0.4 ± 0.8 (range: 0 – 4, median 0, IQR: 0 – 0.75)) and G_I_without_ (4 of 83 recommended measures; on average 0.1 ± 0.3 (range: 0 – 1, median 0, IQR: 0 – 0)) (*P* = 0.025; Poisson regression). Patients in G_I_with_ were three times more likely to rate an optimization measure as helpful than patients of G_I_without_ (IRR: 3.5; 95% CI: 1.2—10.3). The number of optimization measures recommended by the GP (Table 3) had no significant effect on this outcome (*P* = 0.167; Poisson regression). In both groups, more than half of the recommended measures were not remembered by the patients (G_I_with_: 64.1%; G_I_without_: 73.5%, *P* = 0.167; χ^2^ test with Yates’ correction; one measure not analyzable).

**Table 3:** Number of recommended and helpful optimization measures

	G_I_with_	G_I_without_	Between-group comparison
Number of patients with at least one recommended optimization measure (% of all patients)	41 (89.1)	29 (76.3)	*P*_χ2 with Yates’ correction_ = 0.202
Median (range) of the number of recommended optimization measures per patient	2.0 (0—10)	1.5 (0—16)	*P*_Mann-Whitney *U* test_ = 0.268
Total number of helpful optimization measures (% of all optimization measures)	17 (14.5)	4 (4.8)^a^	*P*_χ2 with Yates’ correction_ = 0.052
Number of patients who rated at least one optimization measure as helpful (% of patients)	12 (26.1)	4 (10.5)^b^	*P*_χ2 with Yates’ correction_ = 0.141

G_I_with_: automated and personalized analysis, G_I_without_: exclusively automated analysis

^a^One optimization measure could not be analyzed

^b^One patient could not be analyzed (one optimization measure not rated by patient)

In G_I_with_, the most helpful optimization measures were the recommendations for actions (which were also the most frequently recommended optimization measures in this group; see Additional File 2), such as the recommendation to explain something to the patient or to suggest a spacer for the use of a metered dose inhaler. In G_I_without_ an educational material (e.g. patient leaflet), two times the switch of a product (proposed by an algorithm integrated in the electronic tool) and a measure that was not proposed by the electronic tool were perceived helpful by patients. Indeed, such optimization measures that were not primarily proposed by the electronic tool were recommended most frequently in G_I_without_, e. g. to ensure on the prescription that the drug of one manufacturer cannot be exchanged for a drug of another manufacturer. In G_C_, only two patients reported that they had been recommended one optimization measure each by their GP. The two recommended optimization measures were both considered helpful (sticker for the drug packaging, dose variation to avoid tablet splitting) and both measures would also have been recommended by the tool if it had been applied to the respective BMP.

### GP perception of the benefit of the complexity analysis performed

GP of G_I_with_ expected 39 (84.8%) of their analysis to be helpful for the respective patient. In G_I_without_, significantly fewer analysis, i.e. less than one third (12 analysis; 31.6%) of the analysis were considered helpful by the performing GP (*P* < 0.001; χ^2^ test).

## Discussion

When individual patient preferences were used to personalize an automated analysis, the patients considered significantly more optimization measures helpful than when this was not done. In addition, the GP whose analysis took the patients’ views into account (G_I_with_) considered their analyses to be helpful more than 2.5 times as often as GP only using the automated analysis (G_I_without_). However, although patients and GP in G_I_with_ subjectively valued the results of the intervention, it did not have any influence on the number of medications. The number of complexity factors identified indeed differed in all groups between t_0_ und t_1_, but there was no difference between the three groups, thus the intervention does not seem to have had any influence on this.

Several approaches to reduce medication regimen complexity have been reported [5, 6, 12]. So far, studies evaluating such tools to reduce complexity mostly focused on the reduction of complexity or the number of medications to measure the efficacy of the intervention. In contrast, this study suggests that an intervention was considered helpful by patients without having an impact on the number of complexity factors identified or the number of drugs. This may either indicate that perceived helpfulness of recommendations does not correspond to the objective measurement of outcomes or that complexity alone is not the best indicator of polypharmacy patients’ ability to handle their drug treatment. Indeed, there are complexity factors such as different administration times or splitting tablets that can be resolved easily by changes in the medication regimen [13] which in turn would translate in a reduced complexity score or number of drugs. However, other complexity factors, such as complex dosage forms (e.g. inhalers) can only be mitigated by optimization measures (e.g. training) in most instances. Consequently, these latter optimization measures might be helpful to prevent administration errors, to promote adherence, and, thus, reduce patient-perceived complexity of a medication regimen without reducing complexity scores or the number of drugs. This may be the reason why no statistically significant differences in the number of complexity factors or medication were identified between t_0_ and t_1_ for any group whereas patients and GP still evaluated the intervention positively.

In G_C_ only two patients reported to have talked about complexity in drug treatment with their GP and to have received optimization measures. Thus, this may indicate that GP did not know how to identify complexity of a drug treatment or to reduce complexity in routine care. The results of this study show the potential of an automated analysis that considers the patient perspective to highlight possibilities to reduce the complexity of drug treatment. Nearly nine out of ten patients of G_I_with_ were recommended at least one optimization measure by their GP in this study. And in G_I_without_ three quarters of patients were suggested an optimization measure, even though the GP’s decision to recommend an optimization measure was not necessarily based on the patient perspective.

Given the small number of patients included and optimization measures that were actually remembered by patients at t_1_, the transferability of the results is limited. However, the pilot study presents a rather new approach to focus on the analysis and reduction of complexity from the patients’ point of view. So far, the patients’ perspective has rarely been considered when assessing (or reducing) complexity of drug treatment. In a prospective, observational study a visual analogue scale was used to assess patient’s perception of the complexity of their drug treatment and the results strengthen the idea of considering the patient-perceived complexity of drug treatment, because no concordance was found in the complexity of the antiretroviral therapy perceived by patients and the one calculated using the MRCI [14].

As an exploratory pilot trial, this study has several limitations. First, patients in G_I_without_ had a significantly longer relationship with their GP than patients of the other groups. This might have led to a more trusting relationship with the GP and, thus, it is more likely that difficulties have already been discussed in routine care, which could have weakened the difference between G_I_with_ and G_I_without_. Moreover, also the number of drugs was not equally distributed over the different groups with patients taking on average one drug more in G_I_with_. While the sheer number of drugs does not necessarily lead to a complex drug treatment [3, 15] and in our study, no strong correlation between the number of drugs and the complexity factors that indeed impede the administration of a drug treatment for individual patients was found, it would be helpful to balance further studies with regard to the number of drugs. Additionally, patients were included non-consecutively by GP and, thus, there is a risk of selection bias. Moreover, no further information was collected on patients' health (e.g., current health status) and their current treatment (e.g., frequency of GP visits, previous hospitalization), as these aspects could also influence which optimization measures the GP proposed. Furthermore, the evaluation whether an optimization measure was helpful or not was solely based on the patients’ answers and there was a high percentage of optimization measures that could not be remembered by the patients in the second analysis, although the optimization measures were brought forward directly by the study assistant. One reason could be that the interval between the first and the second analysis was longer than intended (i.e. 6–7 weeks on average instead of 4 weeks as planned) because the new European General Data Protection Regulation (GDPR) became effective which led to a revision of the study documents, and practices and patients were on holidays due to summer months. Furthermore, there could have been optimization measures, e.g. the search for alternative dosage forms, that were performed by the GP without any patient participation. However, all groups were equally affected by the above-mentioned circumstances and, thus, this might not have had an impact on the intervention effect.

## Conclusion

This pilot study suggests that the involvement of patients in the analysis and reduction of the complexity of a drug treatment leads to a needs-based assistance by GP, because patients considered more recommendations by their GP helpful if the analysis also took into account patients’ views. However, the observed effects are small and could have been influenced by different factors, e.g., differences between study groups or a selection bias due to the non-consecutive inclusion of patients.

## Supplementary Information

**Additional file 1.** Histograms that show the distribution of the number of complexity factors and the number of drugs among all three study groups.

**Additional file 2.** Overview of the optimization measures that were considered as helpful for data analysis. The complexity factors identified as relevant for patients, thus leading to the proposal of optimization measures, and a description of the optimization measures suggested by the tool are given.

## Data Availability

The datasets generated and/or analyzed during the current study are not publicly available due to ethical restrictions imposed by the ethics committees of the Medical Faculty of Heidelberg (S-672/2017), the University of Witten/Herdecke (57/2018), the Medical Faculty of Heinrich Heine University Düsseldorf (2018–41-Zweitvotum), the Medical Faculty of University of Rostock (B-F-2018–016), and the Medical Association of Baden Württemberg (A2018-0036), in particular because this potential use case was not included in the patients’ and general practitioners’ informed consent forms, but are available in an anonymized form from the corresponding author on reasonable request.

## Abbreviations

BMP: Nationally standardized medication schedule (“Bundeseinheitlicher Medikationsplan”)
G_C_: Control group (routine care)
G_I_with_: Intervention group (automated and personalized analysis)
G_I_without_: Intervention group (exclusively automated analysis)
GP: General practitioners
MRCI: Medication Regimen Complexity Index
