# Supplementary material for: HIOPP-6 – a pilot study on the evaluation of an electronic tool to assess and reduce the complexity of drug treatment considering patients’ views
Source: BMC Prim Care. 2022 Jun 28;23:164. doi: 10.1186/s12875-022-01757-0 (PMC9241250; doi:10.1186/s12875-022-01757-0)
Supplement: Supplementary file 1 — Additional file 1. Histograms that show the distribution of the number of complexity factors and the number of drugs among all three study groups. [file 12875_2022_1757_MOESM1_ESM.pdf]

Additional File 1

HIOPP-6 – a pilot study on the evaluation of an electronic tool to assess and reduce the complexity of drug treatment considering patients' views

Viktoria S. Wurmbach[1,2]*; Steffen J. Schmidt[3]*; Anette Lampert[1,2]; Simone Bernard[3]; Andreas D. Meid[1]; Eduard Frick[1]; Michael Metzner[1]; Stefan Wilm[4]; Achim Mortsiefer[5,6]; Bettina Bücker[4]; Attila Altiner[7]; Lisa Sparenberg[7]; Joachim Szecsenyi[8]; Frank Peters-Klimm[8]; Petra Kaufmann-Kolle[9]; Petra A. Thürmann[3,10]; Hanna M. Seidling[1,2]**; Walter E. Haefeli[1,2]**

** and ** Both authors contributed equally to the work*

[1] Department of Clinical Pharmacology and Pharmacoepidemiology, Heidelberg University Hospital, Im Neuenheimer Feld 410, 69120 Heidelberg, Germany

[2] Cooperation Unit Clinical Pharmacy, Heidelberg University Hospital, Im Neuenheimer Feld 410, 69120 Heidelberg, Germany

[3] Department of Clinical Pharmacology, School of Medicine, Faculty of Health, Witten/Herdecke University, Alfred-Herrhausen-Straße 50, 58448 Witten, Germany

[4] Institute of General Practice (ifam), Centre for Health and Society (chs), Medical Faculty, Heinrich Heine University Düsseldorf, Moorenstr. 5, 40225 Düsseldorf, Germany

[5] Institute of General Practice (ifam), Centre for Health and Society (chs), Medical Faculty, Heinrich Heine University Düsseldorf, Moorenstr. 5, 40225 Düsseldorf, Germany (affiliation during study conduct)

[6] Professorship of Primary Care, Faculty of Health, Witten/Herdecke University, Alfred-Herrhausen-Straße 50, 58448 Witten, Germany (current affiliation)

[7] Institute of General Practice, Rostock University Medical Center, Doberaner Straße 142, 18057 Rostock, Germany

[8] Department of General Practice and Health Services Research, Heidelberg University Hospital, Im Neuenheimer Feld 130.3, 69120 Heidelberg, Germany

[9] AQUA-Institute for Applied Quality Improvement and Research in Health Care, Maschmühlenweg 8–10, 37073 Göttingen, Germany

[10] Philipp Klee-Institute for Clinical Pharmacology, HELIOS Clinic Wuppertal, Heusnerstraße 40, 42283 Wuppertal, Germany

Corresponding author

PD Dr. sc. hum. Hanna M. Seidling
University of Heidelberg
Department of Clinical Pharmacology and Pharmacoepidemiology
Cooperation Unit Clinical Pharmacy
Im Neuenheimer Feld 410, 69120 Heidelberg, Germany
E-mail: hanna.seidling@med.uni-heidelberg.de
Telephone: + 49 6221 56-38736

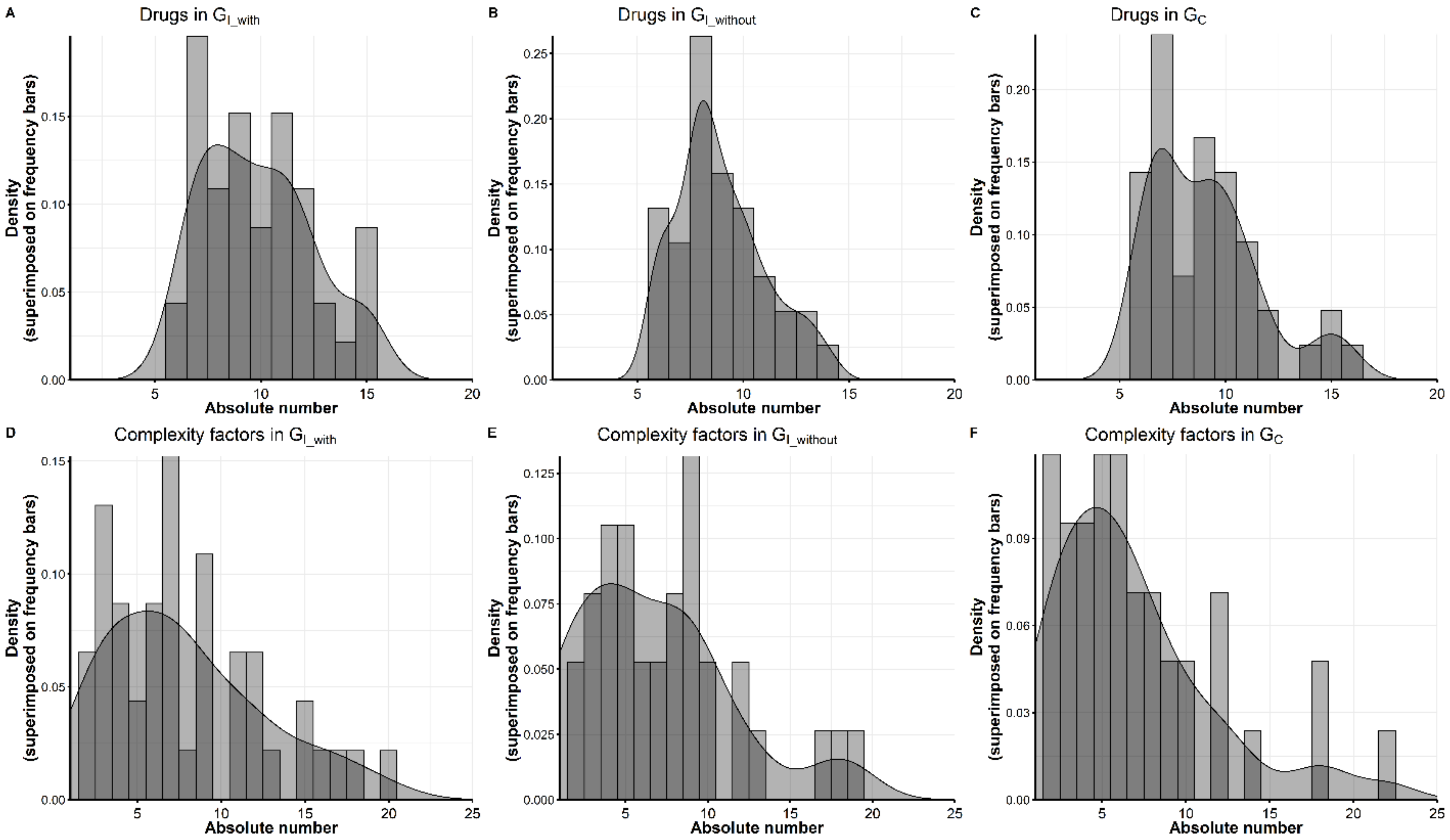

A: Number of drugs in G_{I_with}, B: Number of drugs in $G_{I\_without}$, C: Number of drugs in Gc, D: Number of complexity factors in G_{I_with}, E: Number of complexity factors in $G_{I\_without}$, F: Number of complexity factors in G_C

G_{I_with}: intervention group (automated and personalized analysis), $G_{I\_without}$: intervention group (exclusively automated analysis),
G_C: control group (routine care)
