# Supplementary material for: HIOPP-6 – a pilot study on the evaluation of an electronic tool to assess and reduce the complexity of drug treatment considering patients’ views
Source: BMC Prim Care. 2022 Jun 28;23:164. doi: 10.1186/s12875-022-01757-0 (PMC9241250; doi:10.1186/s12875-022-01757-0)
Supplement: Supplementary file 2 — Additional file 2. Overview of the optimization measures that were considered as helpful for data analysis. The complexity factors identified as relevant for patients, thus leading to the proposal of optimization measures, and a description of the optimization measures suggested by the tool are given. [file 12875_2022_1757_MOESM2_ESM.pdf]

Additional File 2

HIOPP-6 – a pilot study on the evaluation of an electronic tool to assess and reduce the complexity of drug treatment considering patients' views

Viktoria S. Wurmbach[1,2]*; Steffen J. Schmidt[3]*; Anette Lampert[1,2]; Simone Bernard[3]; Andreas D. Meid[1]; Eduard Frick[1]; Michael Metzner[1]; Stefan Wilm[4]; Achim Mortsiefer[5,6]; Bettina Bücker[4]; Attila Altiner[7]; Lisa Sparenberg[7]; Joachim Szecsenyi[8]; Frank Peters-Klimm[8]; Petra Kaufmann-Kolle[9]; Petra A. Thürmann[3,10]; Hanna M. Seidling[1,2]**; Walter E. Haefeli[1,2]**

** and ** Both authors contributed equally to the work*

[1] Department of Clinical Pharmacology and Pharmacoepidemiology, Heidelberg University Hospital, Im Neuenheimer Feld 410, 69120 Heidelberg, Germany

[2] Cooperation Unit Clinical Pharmacy, Heidelberg University Hospital, Im Neuenheimer Feld 410, 69120 Heidelberg, Germany

[3] Department of Clinical Pharmacology, School of Medicine, Faculty of Health, Witten/Herdecke University, Alfred-Herrhausen-Straße 50, 58448 Witten, Germany

[4] Institute of General Practice (ifam), Centre for Health and Society (chs), Medical Faculty, Heinrich Heine University Düsseldorf, Moorenstr. 5, 40225 Düsseldorf, Germany

[5] Institute of General Practice (ifam), Centre for Health and Society (chs), Medical Faculty, Heinrich Heine University Düsseldorf, Moorenstr. 5, 40225 Düsseldorf, Germany (affiliation during study conduct)

[6] Professorship of Primary Care, Faculty of Health, Witten/Herdecke University, Alfred-Herrhausen-Straße 50, 58448 Witten, Germany (current affiliation)

[7] Institute of General Practice, Rostock University Medical Center, Doberaner Straße 142, 18057 Rostock, Germany

[8] Department of General Practice and Health Services Research, Heidelberg University Hospital, Im Neuenheimer Feld 130.3, 69120 Heidelberg, Germany

[9] AQUA-Institute for Applied Quality Improvement and Research in Health Care, Maschmühlenweg 8–10, 37073 Göttingen, Germany

[10] Philipp Klee-Institute for Clinical Pharmacology, HELIOS Clinic Wuppertal, Heusnerstraße 40, 42283 Wuppertal, Germany

Corresponding author
PD Dr. sc. hum. Hanna M. Seidling
University of Heidelberg
Department of Clinical Pharmacology and Pharmacoepidemiology
Cooperation Unit Clinical Pharmacy
Im Neuenheimer Feld 410, 69120 Heidelberg, Germany
E-mail: hanna.seidling@med.uni-heidelberg.de
Telephone: + 49 6221 56-38736

Table 1 Overview of optimization measures that were rated helpful by patients

Complexity factor	Optimization measure proposed by GP*	Description of optimization measure	Evaluation of optimization measure by patient (N)	Number of times (N = number of times G_{I_with} affected)
**Complexity factors identified by automated assessment**				
Tablet splitting	Exchange of medication	Algorithm that suggests preparations in the appropriate strength, so that sharing is not necessary	improved (2); little improved (1)	3 (1)
Medication on demand	Recommendations for actions	Recommendation to explain the use of this medication (e.g. the symptoms for which it should be used, the dose recommended) and to add the information to the nationally standardized medication schedule	little improved (1)	1 (1)
	Other measure	Chosen by GP, i.e. not proposed by electronic tool	improved (1)	1 (0)
Administration at lunch time	Recommendations for actions	Recommendation for GP to check whether the use of the drug can be changed to another point in time	improved (1)	1 (1)
Once weekly administration	Recommendations for actions	Recommendation of different tools and measures to organize drug treatment (e.g. medication dispenser, app for smartphones, setting an alarm clock)	little improved (1)	1 (1)
Only one drug at one specific point in time	Recommendations for actions	Recommendation for GP to check whether the use of the drug can be changed to another point in time	little improved (1)	1 (1)

Potentially increased need for training in dosage form use	Training material	Patient leaflets on the use of different dosage forms were provided	little improved (1)	1 (0)
Potentially patient-unfriendly nature of liquid oral dosage forms	Recommendations for actions	Recommendation for GP to check whether an alternative preparation or dosage form would be reasonable	improved (1)	1 (1)
**Complexity factors identified by additional questions (non-automated)**				
No use of medication schedule	Recommendations for actions	Recommendation to explain of design, use and benefits of nationally standardized medication schedule	improved (3);<br>little improved (2)	5 (5)
Cognitive impairment	Recommendations for actions	Recommendation of different tools and measures to organize drug treatment (e.g. medication dispenser, appfor smartphones, setting an alarm clock)	improved (2);<br>little improved (1)	3 (3)
Changes in existing medication regimen, New prescription, Frequent generic substitution, Changes in tablet color or shape, Hospital discharge	Recommendations for actions**	Recommendation for GP to check whether the difficulties can be avoided by marking the preparation or adding explanations on the medication schedule or changing the preparation	little improved (1)	1 (1)
Intricate packaging	Recommendations for actions**	Recommendation for GP to discuss the problems and choose alternative preparations	little improved (1)	1 (1)
Swallowing difficulties	Recommendations for actions	Recommendation of several aids that can help swallow a drug (e.g. additional film coatings)	improved (1)	1 (1)

G_{I_with}: automated and personalized analysis; GP: general practitioners

*GP logged the optimization measures they proposed in the tool, for several complexity factors two or three different optimization measurers were suggested by the tool; **GP logged in the electronic tool that they proposed a measure in addition to the recommendation for action, that was not suggested by the tool, but patients could not remember any other optimization measure
